# From eHealth to iHealth: Transition to Participatory and Personalized Medicine in Mental Health

**DOI:** 10.2196/jmir.7412

**Published:** 2018-01-03

**Authors:** Sofian Berrouiguet, Mercedes M Perez-Rodriguez, Mark Larsen, Enrique Baca-García, Philippe Courtet, Maria Oquendo

**Affiliations:** ^1^ Lab-STICC IMT Atlantique Université Bretagne Loire Brest France; ^2^ Laboratoire Soins primaires Santé publique, Registre des cancers de Bretagne Occidentale SPURBO Equipe d'accueil 7479 Brest France; ^3^ Department of Psychiatry Icahn School of Medicine at Mount Sinai New York, NY United States; ^4^ Black Dog Institute University of New South Wales Sydney Australia; ^5^ Department of Psychiatry Fundación Jimenez Diaz Hospital Autónoma University, Centro de Investigacion en Red Salud Mental Madrid Spain; ^6^ Department of Emergency Psychiatry University Hospital of Montpellier University of Montpellier Montpellier France; ^7^ Perelman School of Medicine University of Pennsylvania Philadelphia, PA United States

**Keywords:** data mining, decision making, mobile phone, Web app, mental health

## Abstract

Clinical assessment in psychiatry is commonly based on findings from brief, regularly scheduled in-person appointments. Although critically important, this approach reduces assessment to cross-sectional observations that miss essential information about disease course. The mental health provider makes all medical decisions based on this limited information. Thanks to recent technological advances such as mobile phones and other personal devices, electronic health (eHealth) data collection strategies now can provide access to real-time patient self-report data during the interval between visits. Since mobile phones are generally kept on at all times and carried everywhere, they are an ideal platform for the broad implementation of ecological momentary assessment technology. Integration of these tools into medical practice has heralded the eHealth era. Intelligent health (iHealth) further builds on and expands eHealth by adding novel built-in data analysis approaches based on (1) incorporation of new technologies into clinical practice to enhance real-time self-monitoring, (2) extension of assessment to the patient’s environment including caregivers, and (3) data processing using data mining to support medical decision making and personalized medicine. This will shift mental health care from a reactive to a proactive and personalized discipline.

## Introduction

### Evolution From eHealth to iHealth

Clinical assessment in psychiatry is usually based on findings from brief, regularly scheduled in-person appointments. Although critically important, this approach reduces assessment to cross-sectional observations that miss essential information about disease course and are subject to recall bias. The mental health provider makes all medical decisions based on this limited information.

After an initial longer assessment, standard follow-up outpatient visits in a psychiatric clinic usually include a mental status examination, a brief update on the history of the patient’s present illness (including a safety assessment of risk of self-harm, suicide, and homicide), an assessment of treatment effectiveness and potential side effects, and an updated diagnostic impression and treatment plan. All of this generally happens in under 30 minutes. Except in patients with severe mental illness or disabilities, family members or caregivers rarely attend these visits.

As the duration of a psychiatric outpatient visit becomes increasingly shorter and intervals between appointments longer, it is essential to develop a form of assessment that can more accurately track patients’ symptoms between visits [1]. One possible solution is to use personal health records (PHRs), longitudinal health records self-reported by the patient. PHRs can be based on mobile devices (mobile phones, wearable devices) or Web-based self-monitoring. Validity, reliability, and acceptability of this online approach is similar to traditional paper-pencil questionnaires in mental health patients [2]. Surprisingly, although studies have highlighted the value of patient self-reports in clinical assessment, they rarely are routinely implemented [3]. This is despite the fact that many commercial electronic health record (EHR) software packages already allow data entry by patients and caregivers.

Over the last decade, medical assessments have been supported by the increasing use and importance of EHRs that facilitate the portability of pertinent health information across providers and geographic locations. Interinstitutional EHRs further increase efficiency in medical services and provide complete and accurate medical information across providers in different institutions [4]. However, their implementation has only had a modest impact on clinical outcomes and measures of quality of care [5].

Internet features have increased networking possibilities of EHRs, offering new options for patient monitoring. Integration of these tools into medical practice has heralded the electronic health (eHealth) era, integrating new technologies into routine clinical practice. eHealth can also support patient self-monitoring, where both patient and caregivers can update a log of the patient’s mental and physical state between medical visits, potentially leading to more accurate assessment. For example, eHealth tools allow an endocrinologist to chart blood glucose levels between visits for a patient with a portable blood glucose meter. Similarly, a mental health clinician could have access to, and be able to chart, changes in sleep, mood, appetite, and other relevant data related to illness course between visits. These data can be collected through Web-based or mobile phone–based self-reports and other assessment tools and sensors. The clinician can take these data into account during the clinical decision-making process before or during the in-person visit.

**Figure 1:**
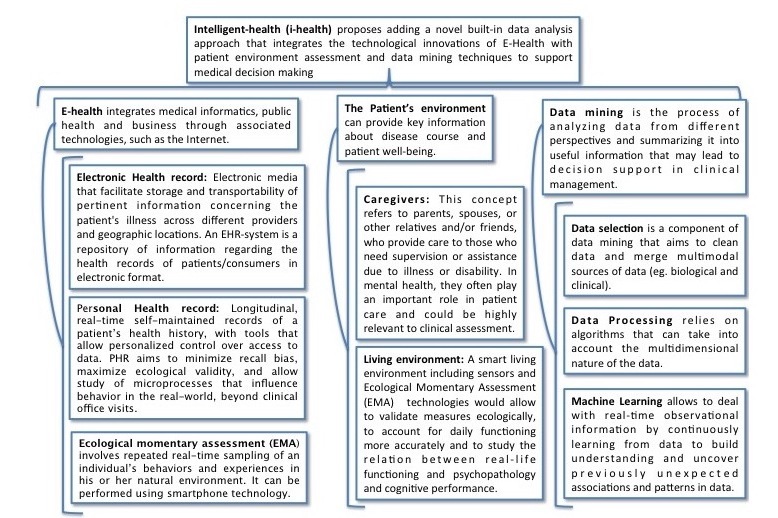
Terms and definition related to e-health.

**Figure 2:**
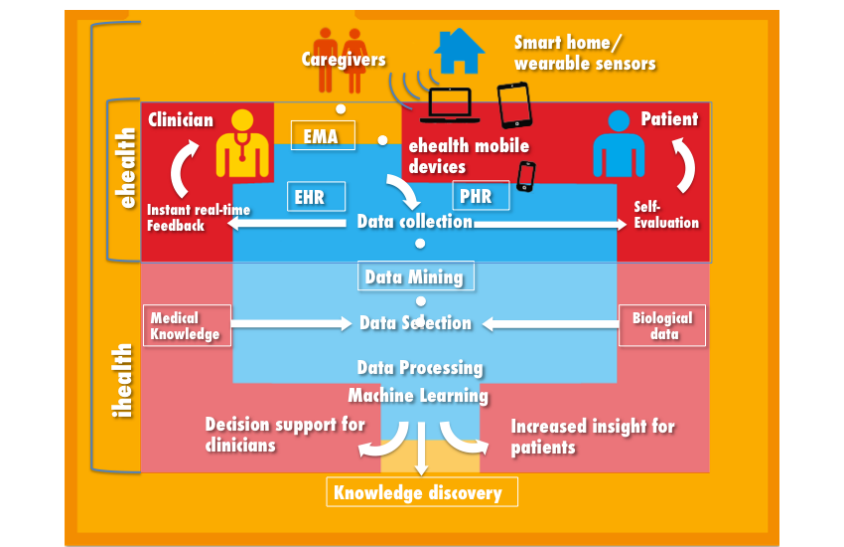
The transition from electronic health (eHealth) to intelligent health (iHealth). EMA: ecological momentary assessment; EHR: electronic health record; PHR: personal health record.

However, we need to go beyond eHealth and move to intelligent health (iHealth). iHealth further builds on and expands eHealth by combining real-time self-monitoring with more contextual information from the patient’s environment and novel built-in data analysis tools to enhance medical decision making. The transition from eHealth to iHealth will require integration of comprehensive data from the patient’s environment, as reported by the patient or caregiver or captured through sensors in the patient’s living environment, and the use of artificial intelligence data-mining techniques to aid clinical decision making and provide more personalized treatment (Figures 1 and 2).

iHealth will allow a mental health provider to receive real-time input from data-mining tools that will help guide clinical decision making. This is particularly important in the field of psychiatry, where the lack of biomarkers and objective biological tests means that most clinical decisions (eg, diagnosis, treatment choice, admission and discharge, risk stratification) are based on signs, symptoms, and behaviors reported to or directly observed by the clinician during the clinical interview. For example, a data-mining iHealth tool may generate a message to alert a clinician that a patient diagnosed with bipolar I disorder is showing a pattern of decreased sleep and increased activity that suggests the imminence of a manic episode. The clinician could decide, based on the alert and other available information, to have a member of the treatment team contact the patient to assess mood stability and determine whether a treatment change is warranted.

In developing countries, iHealth could be used to increase access to specialized care for underserved populations. For example, a data-mining tool could generate data-driven personalized treatment recommendations for a given patient that would be implemented by a general practitioner [6].

Below, we outline a novel iHealth model for clinical assessment and treatment in psychiatry based on (1) incorporation of new technologies into clinical practice to enhance real-time self-monitoring, (2) extension of assessment to the patient’s environment, and (3) data processing using data mining to support medical decision making and personalized medicine.

## Emerging Treatment Models

### eHealth Era in Mental Health: From Electronic Health Records to Ecological Momentary Assessment

By the end of 2014, there were almost 3 billion Internet users, two-thirds from developing countries, and mobile-broadband subscriptions reached 2.3 billion globally [7]. With such technological advances and reach it is already possible to incorporate Web-based and mobile phone apps into clinical assessment and treatment. Given that psychiatry clinicians have previously relied exclusively on clinical interviews for diagnosis and treatment, the field could deeply benefit from this new source of data collected in real time covering information about the patient’s health state between visits. Mobile phones are generally kept on at all times and carried everywhere, making them an ideal platform for the broad implementation of ecological momentary assessment (EMA) technology. EMA involves repeated sampling of subjects’ behaviors and experiences in real time, in their natural environment. Patient self-monitoring can rely on EMA procedures and lead to participatory medicine. EMA has been successfully used for real-time self-reporting of symptoms and behavior—for example, Husky et al [8] showed the utility and feasibility of using EMAs to study suicidal ideation [8].

The emergence of smart homes and the development of sensor technologies allows the nonintrusive collection of activity data [9], allowing objective analysis of an individual’s behavioral patterns. Thus, health-related events such as activities of daily living (ADLs, feeding, taking care of one’s personal hygiene, dressing) can be captured without the patient’s active participation. Monitoring behavioral patterns of psychiatric patients and their ability to carry out their ADLs in their living environment will likely improve knowledge about disease course. The detection of changes in patterns of behavior may help to detect emerging disorders [10] and study the relationship between functioning and cognitive performance or illness course [11]. In mental health, this approach is still at an early stage but has already shown promising results in the monitoring of depressive symptoms in cognitively impaired patients [12] and of activity in patients with schizophrenia [13]. Smart Home and Ambient Assisted Living (SHAAL) systems use sensors and other devices that are either wearable or integrated in the patient’s home and have been used to assess the effect of negative symptoms and cognitive impairment on ADL functions [14] and detect emerging disorders based on changes in the patient’s behavior [15].

Future studies need to assess the impact of the support provided by these smart home devices on patient outcomes and the sensitivity and specificity of the data collection devices. The cost-effectiveness of these new monitoring approaches also needs to be assessed. Furthermore, the ethical and legal aspects have to be addressed, taking into account privacy and medical confidentiality issues [16].

### Including Patient Environment in Mental Health Monitoring

Many psychiatric disorders are chronic illnesses associated with high levels of disability [17], making it a challenge for some psychiatric patients to live independently. Therefore, caregivers often play a critical role in the lives of those with serious mental illness [18].

Studies have shown that caregivers and close contacts are reliable sources of information about patients with psychiatric disorders [19]. Traditional psychiatric assessment, however, does not always include information from caregivers due to time constraints and concerns about confidentiality [20]. By excluding caregivers from assessments, clinicians may miss an opportunity to obtain additional valuable information about the illness course [21].

The new technologies described above, including PHRs and EMA, can easily include not only the patient’s own reports and activity but also those from a caregiver [22]. Capturing caregivers’ reports through an EMA approach could provide a more accurate assessment of the illness course in a given patient. Both the patient and the caregiver would be able to enter updates in an online log of the patient’s mental and physical state between outpatient visits.

Involving caregivers in the clinical assessment may also help to decrease the caregiver burden, including physical and psychological stress, social pressure, and financial costs associated with care giving. Unfortunately, studies focusing on caregiver burden are scarce and have used small heterogeneous patient samples [23]. Identifying and modifying caregivers’ burdens through EMA might help to reduce the level of burden and its negative effects, both on the caregivers and on patient outcomes [20].

We are advancing toward a double paradigm shift. First, the integration of patient and caregiver data through EMA and PHRs into routine psychiatric assessment will give clinicians access to real-time reports and behaviors of patients and caregivers. Second, as described in the next section, the application of data-mining techniques to the EMA and PHR data will support and enhance medical decision making. These data can be analyzed using data mining tools in order to develop predictive models and personalized treatments. This will shift mental health from a reactive to a proactive discipline, leading to decision support systems for clinicians, as summarized in Figure 1.

### Transforming Data into Knowledge Through iHealth

The traditional method of turning data into knowledge has relied on manual data analysis and interpretation of results in order to find useful patterns to support decision making. The enormous amount and complexity of the ecological data that can be obtained through EMA and PHRs [24,25] make manual analysis by end users (that is, clinicians) difficult or impossible. Making sense of enormous datasets is a common challenge in the Big Data era that is best overcome using data mining techniques.

**Figure 3:**
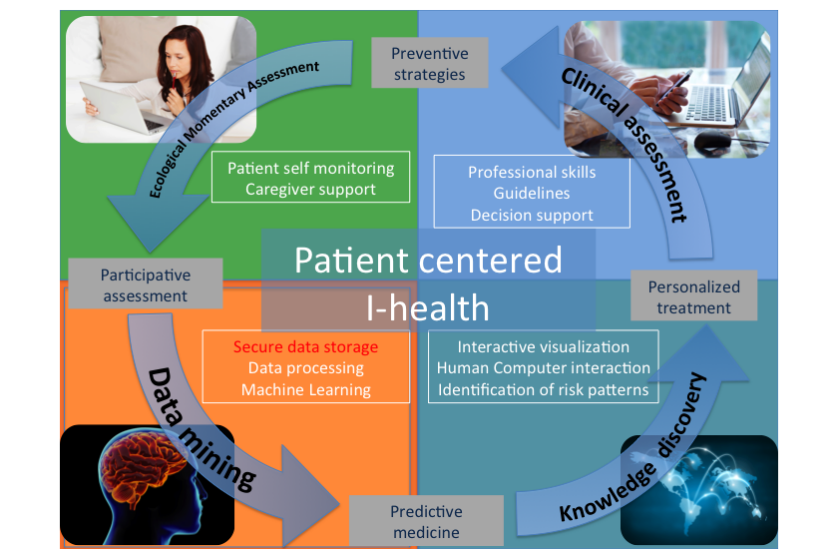
iHealth in the context of the 4P (predictive, personalized, preventive and participatory) model of mental health.

Data mining is a set of techniques that can be used to explore treatment and outcome questions in large clinical databases and help develop algorithms and guidelines for problems where controlled data are difficult to obtain. The data mining process includes several steps, including data selection, data processing, and machine learning (described below). Data mining techniques can be used to find relationships and patterns between EMA and PHR data and neurobiological data. As an example, experiments have shown that connections between momentary mental states and environments are sensitive to genetic effects, emphasizing gene-environment interaction [26]. This may lead to advances in clinical decision making, incorporating clinical, ecological, and biological data [27,28].

Machine learning techniques seek to answer the question: How can we build computer systems that automatically improve with experience, and what are the fundamental laws that govern all learning processes? Machine learning techniques allow processing of real-time observational information by continuously learning from data to build understanding and uncover previously unexpected associations and patterns in data [29]. Predictive and explanatory models might use individual patient data to predict future events like the probability of a patient attempting suicide in a given time interval. Machine learning models are continuously updated to refine and improve their clinical applicability [30]. This process may significantly improve decision making [31] and knowledge discovery (Figures 2 and 3). Specifically, data mining allows for the exploration of risk factors, patterns of symptom evolution, and identification of high-risk subgroups [32].

## Discussion

### iHealth as a Practical and Conceptual Challenge for Mental Health Professionals and Patients

Despite its many potential advantages over traditionally used psychiatric assessment tools, iHealth still faces several risks and challenges related to human factors. First, electronic monitoring devices are often experienced by physicians as financial, technical, or time-consuming threats. Furthermore, psychological barriers are also important to consider [33]. With the implementation of iHealth tools, physicians may be concerned about the loss of control over patient information and the decision-making process, since these data can be shared with and assessed by others. It is uncertain if clinicians will be willing to share the decision-making process with machine-learning tools and a data-analysis team.

It could be argued that eHealth and iHealth may be most useful for severe and disabling psychiatric disorders, which are characterized by poor insight and often require significant involvement of caregivers and family members in the clinical care. Some examples could be major neurocognitive disorders (dementia), severe psychotic disorders (schizophrenia), or autism spectrum disorders. However, it should be noted that growing evidence suggests that family involvement and knowledge about the illness and regular contact between patients and caregivers improve outcomes across psychiatric disorders [34-36].

Another limitation of iHealth is that it has been argued that some psychiatric conditions are not amenable to self-reporting [37]. If a patient is in crisis or suffers from cognitive impairment or psychosis, EMA or PHR assessments may be of limited utility. In such situations, however, sensor-based technologies can still provide valuable objective data [6].

It may also be argued that requiring the patient to constantly keep track of their mental and physical state puts more responsibility and burden on them. Whether this would have positive or negative effects remains unexplored [38]. eHealth and iHealth interventions require an important time commitment from the participants, particularly for those who collect regular daily EMA data. This approach may be significantly more invasive than asking a participant to complete a retrospective questionnaire or answer a question through a traditional interview. The risk of intrusiveness into daily lives exists, but this issue was not assessed in the articles we reviewed or in other reviews in the field. While a growing number of health care systems in developed countries use some form of EHR or PHR, very few use eHealth for the delivery of mental health care. Therefore, increasing the update of eHealth interventions is a crucial step toward realizing the potential of iHealth.

Data privacy concerns may deter patients from sharing personal data related to mental health, but data security procedures have been a routine part of data mining from the outset [39]. Human factors including acceptability aspects regarding the technology and control over personal data will be critical to accomplish the transition to iHealth.

### Conclusion

Despite the challenges and limitations iHealth may lead to improved clinically integrated decision-making tools and personalized medicine practice, tailoring medical treatment to each individual patient. Building on the advances in mental health assessment brought about by eHealth, iHealth will provide personalized clinical information outside of clinical visits and integration of real-time multimodal patient and caregiver data using data mining technologies. This will allow for more precise and effective clinical assessment and decision making. The ability to mine large databases for new hypotheses regarding clinical and environmental dynamic patterns of psychiatric illness through iHealth could also change clinical practice. These possibilities serve key public health needs and offer intriguing and novel opportunities for collaboration, knowledge production, and data analysis.

## Abbreviations

ADL: activity of daily living
eHealth: electronic health
EHR: electronic health record
EMA: ecological momentary assessment
iHealth: intelligent health
PHR: personal health record
SHAAL: Smart Home and Ambient Assisted Living
